# Lipoprotein(a) as a novel biomarker for predicting adverse outcomes in ischemic heart failure

**DOI:** 10.3389/fcvm.2024.1466146

**Published:** 2024-09-05

**Authors:** Biyang Zhang, Yinxiao Xu, Xin Huang, Tienan Sun, Meishi Ma, Zheng Chen, Yujie Zhou

**Affiliations:** Department of Cardiology, Beijing Anzhen Hospital, Capital Medical University, Beijing, China

**Keywords:** lipoprotein(a), ischemic heart failure, major adverse cardiovascular events, body mass index, restricted cubic spline (RCS) curves

## Abstract

**Background:**

Lipoprotein(a) [Lp(a)] is an independent risk factor for atherosclerotic cardiovascular disease (ASCVD). However, the association between Lp(a) and adverse outcomes in patients with ischemic heart failure (IHF) remains unclear. This study aimed to investigate the relationship between serum Lp(a) levels and the incidence of major adverse cardiovascular events (MACE) in IHF patients.

**Methods:**

In this single-center, retrospective cohort study, 1,168 IHF patients who underwent elective percutaneous coronary intervention (PCI) were enrolled. Patients were divided into four groups based on Lp(a) quartiles. The primary endpoint was MACE, defined as a composite of all-cause mortality, non-fatal myocardial infarction (MI), and any revascularization. Cox proportional hazards models were used to evaluate the association between Lp(a) quartiles and adverse outcomes. Restricted cubic spline (RCS) curve were constructed to explore the nonlinear relationship between Lp(a) levels and MACE risk. Subgroup analyses were performed to investigate the association in different subgroups.

**Results:**

The incidence of MACE increased significantly across Lp(a) quartiles (Quartile 4 vs. Quartile 1: 46.4% vs. 22.9%, *P* < 0.001). After adjusting for confounding factors, the highest Lp(a) group remained independently associated with an increased risk of MACE (HR, 95% CI: 2.28, 1.69–3.07, *P* < 0.001, P for trend <0.001), all-cause mortality (HR, 95% CI: 2.33, 1.54–3.54, *P* < 0.001, P for trend = 0.01), and any revascularization (HR, 95% CI: 2.18, 1.35–3.53, *P* = 0.002, P for trend = 0.001). The RCS model demonstrated a nonlinear positive relationship between Lp(a) levels and MACE risk. Subgroup analysis revealed a significant interaction with body mass index (BMI), with a more pronounced association observed in patients with higher BMI (P for interaction <0.001).

**Conclusion:**

Elevated Lp(a) levels were independently associated with an increased risk of MACE, mortality, and revascularization in IHF patients, with a stronger effect in obese individuals.

## 1 Introduction

The prevalence of heart failure, a global public health concern, is increasing due to an aging population (1, 2). It is estimated that there are approximately 26 million individuals with heart failure worldwide, and this figure is projected to rise in the forthcoming decades (3). Heart failure not only impacts patients’ well-being but also places a significant strain on healthcare systems. Although the etiology of heart failure varies by region, ischemic heart failure (IHF) is the primary cause of this condition globally, especially in low-income countries (4–6). In fact, in many low-income countries, IHF accounts for more than 50% of heart failure cases (5). Moreover, compared to other etiologies, patients with IHF have a worse prognosis and higher one-year mortality (5). Therefore, enhancing the prognosis of patients with IHF is a critical matter that requires urgent attention.

Lipoprotein(a), abbreviated as Lp(a), consists of a complex structure comprising apolipoprotein(a) and low-density lipoprotein (LDL). The liver is the primary site for Lp(a) synthesis, and genetic factors predominantly determine Lp(a) concentrations, while diet, age, and environmental factors have minimal impact (7, 8). Recent studies have indicated that Lp(a) may promote atherosclerosis development through various mechanisms and serves as a standalone risk factor for atherosclerotic cardiovascular disease (ASCVD) independent of conventional risk factors (9). Extensive epidemiological research has established a strong link between high Lp(a) concentrations and a greater likelihood of developing several cardiovascular disorders, such as coronary artery disease (CAD), stroke, and aortic valve stenosis (10–13). Furthermore, prior investigations have shown that increased Lp(a) levels were linked to unfavorable long-term cardiovascular outcomes in CAD patients following percutaneous coronary intervention (PCI) (14–19).

Nonetheless, limited studies have explored the significance of Lp(a) in individuals with IHF (10). Given the strong association between Lp(a) and atherosclerosis, as well as adverse cardiovascular outcomes, coupled with the pivotal role of atherosclerosis in the pathogenesis of IHF, we hypothesized that Lp(a) might serve as a significant risk factor for patients with IHF. Hence, the objective of this study was to examine the relationship between serum Lp(a) concentrations and the occurrence of major adverse cardiovascular events (MACE) in IHF patients.

## 2 Methods

### 2.1 Study population

**Figure 1:**
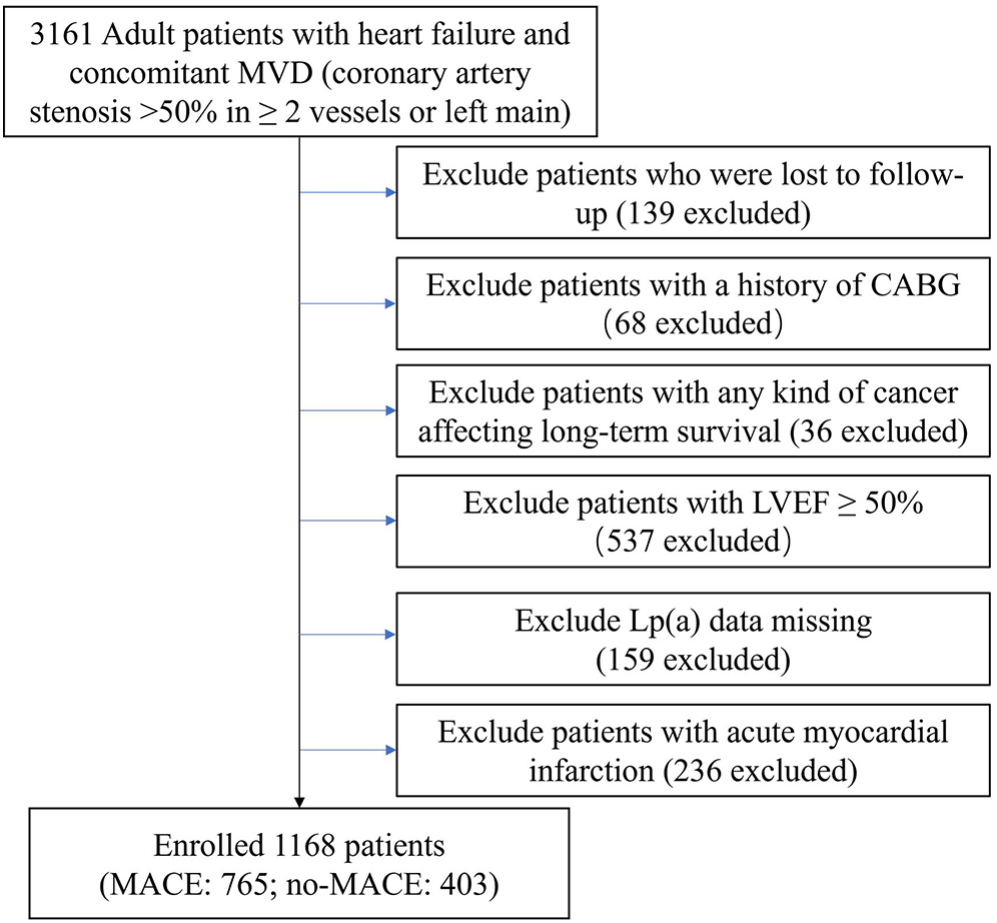
Flow chart of study population.

In this single-center, retrospective cohort study, we examined patients diagnosed with IHF who underwent elective PCI at Beijing Anzhen Hospital from June 2017 to June 2019. HF was diagnosed according to specific criteria (20), including heart failure as per ICD-10 and concurrent multivessel CAD with >50% narrowing of 2 or more coronary arteries or the left main. Our cardiac center initially enrolled 3,161 patients. After applying the exclusion criteria, the final analysis comprised 1,963 patients (Figure 1). The study received approval from the hospital's ethics board (code 2022235X).

### 2.2 Data collection

Data on patient characteristics, including demographics, vital signs, and body mass index (BMI), New York Heart Association (NYHA) class, comorbidities (atrial fibrillation, hypertension, diabetes, hypercholesterolemia), medical history (prior stroke, prior myocardial infarction (MI), prior percutaneous coronary intervention (PCI)), laboratory parameters (white blood cell, red blood cell, hemoglobin, platelet, fasting blood glucose (FBG), glycosylated hemoglobin A1c (HbA1c), creatinine, blood nitrogen urea, estimated glomerular filtration rate (eGFR), triglycerides (TG), total cholesterol (TC), low-density lipoprotein cholesterol (LDL-C), high-density lipoprotein cholesterol (HDL-C), sodium, potassium, B-natriuretic peptide (BNP), high sensitivity C-reactive protein (hs-CRP)), echocardiography (left atrial diameter, left ventricular end-systolic diameter (LVDs), left ventricular end-diastolic diameter (LVDd), left ventricular ejection fraction (LVEF)), medication use (antiplatelet agents, lipid-lowering drugs, antihypertensive medications, and diuretics), angiographic findings (coronary artery disease severity, lesion characteristics, and SYNTAX score), and procedural outcomes (treated vessels, completeness of revascularization, and number of stents implanted) were obtained from the institution's electronic medical records system. The SYNTAX score was determined using the official SYNTAX score calculator (www.syntaxscore.com). Angiographic images were assessed by a minimum of two experienced cardiologists. Lp(a) levels were measured using Latex-Enhanced Immunoturbidimetry (LEIT). All data were collected within the first 24 h after patient admission or after the completion of PCI.

### 2.3 Grouping and outcomes

Researchers categorized study subjects into quartiles according to their Lp(a) levels (mg/dl): Group 1: Lp(a) < 6.5 (*n* = 292), Group 2: 6.5 ≤ Lp(a) < 15 (*n* = 293), Group 3: 15 ≤ Lp(a) < 33 (*n* = 291), and Group 4: Lp(a) ≥ 33 (*n* = 291). MACE, the primary outcome, included death from any cause, non-fatal MI, and any revascularization procedures. The individual components of MACE were considered as secondary endpoints. Non-fatal MI was defined as an MI that did not directly result in the patient's death. According to the Fourth Universal Definition of MI (21), MI was characterized by an elevation in troponin levels, with at least one value exceeding the 99th percentile of the upper reference limit, accompanied by one or more of the following criteria: (1) symptoms indicative of myocardial ischemia, (2) new ischemic changes observed on ECG, (3) development of pathological Q waves, (4) imaging evidence of new viable myocardial loss or new regional wall motion abnormality consistent with an ischemic etiology, or (5) detection of a coronary artery thrombus via angiography or autopsy.

### 2.4 Follow-up

Post-baseline PCI, skilled staff conducted regular patient follow-ups every 3 months for the first year, and then at 24 and 36 months. Outpatient visits and phone calls with patients or family members provided data on MACE and medications. Hospital readmissions during the follow-up period were also recorded. If a patient had several adverse events, only the most serious one (prioritized in the order of death, heart attack without death, and revascularization) was analyzed. For recurring events, solely the initial occurrence was taken into account. Follow-up for the research ended in mid-2022, covering a 3-year period.

### 2.5 Statistical analysis

The study presented baseline characteristics as mean ± SD for normally distributed continuous variables, median (IQR) for skewed continuous variables, and numbers (%) for categorical variables. One-way ANOVA, Kruskal–Wallis test, and Chi-square test were employed to compare differences among the Lp(a) quartiles for normally distributed, skewed, and categorical variables, respectively.

Cox proportional hazards models were utilized to assess the associations between Lp(a) quartiles and adverse outcomes. Hazard ratios (HR) with 95% confidence intervals (CI) were reported for the results. The lowest quartile of Lp(a) was used as the reference group. Three models were used: Model I (unadjusted), Model II (adjusted for age and sex), and Model III (further adjusted for age, sex, heart rate, BMI, NYHA class, prior PCI, TG, LDL-C, platelet, potassium, complete revascularization, diffuse lesion, chronic total occlusion, SYNTAX score, diffuse lesion, angiotensin receptor blocker (ARB), diuretics, statins. Univariate analysis was performed to identify variables for inclusion Model III. Univariate analysis identified variables (*P* < 0.05) for inclusion in the multivariate Cox proportional hazards model. To address the potential issue of multicollinearity among predictor variables, we employed Variance Inflation Factor (VIF) analysis. Variables exhibiting a VIF exceeding 10 were excluded from Model III. The Schoenfeld Test was used to evaluate the proportional hazards assumption, calculating the Schoenfeld Individual Test *p*-value and the Global Schoenfeld Test *p*-value when Lp(a) was included in the model as both a categorical and a continuous variable. Kaplan-Meier survival analysis and the log-rank test were used to assess and compare the cumulative incidence of adverse outcomes among the Lp(a) quartiles. The study employed Restricted Cubic Spline (RCS) curves, a non-parametric regression method, to examine the potential nonlinear relationship between Lp(a) levels and MACE risk. The analysis was conducted while adjusting for covariates in Model III. A nonlinear relationship was deemed statistically significant if the nonlinear *p*-value was below 0.05. The number of knots was selected based on the minimum value of the Akaike Information Criterion (AIC). The core idea of the AIC is to assess the relative quality of a model on a given dataset, particularly balancing goodness-of-fit and model complexity. In this study, four knots were ultimately chosen to construct the RCS model and plot the curve.

The association between Lp(a) and MACE risk in different subgroups was investigated using univariate Cox proportional hazards models in subgroup analyses. A forest plot was generated, and the interaction *P*-value was calculated.

Statistical analyses were conducted using R software version 4.2.1 (R Foundation for Statistical Computing, Vienna, Austria). The following packages were utilized: “survival”, “ggplot2”, “rms”, “survminer”, and “forestplot”. Statistical significance was set at a two-tailed *P*-value < 0.05.

## 3 Results

### 3.1 Subjects and baseline characteristics

In total, 1,168 patients participated in the current study. Participants were categorized into four groups according to Lp(a) level quartiles. Table 1 summarized the baseline characteristics for each group. Patient age significantly increased across ascending Lp(a) quartiles (*P* = 0.006). Those in upper Lp(a) quartiles exhibited reduced systolic blood pressure (*P* = 0.019) and elevated TC (*P* = 0.001), LDL-C (*P* < 0.001), BNP (*P* = 0.043), and hs-CRP (*P* = 0.048). A significantly greater proportion of patients in the higher Lp(a) quartiles had a history of stroke (*P* = 0.014). Additionally, TG levels were lower in patients in the upper Lp(a) quartiles (*P* = 0.006). However, echocardiography, medication use, and angiography results did not differ significantly between the groups.

**Table 1:** Characteristics of patients stratified by Lp(a) quartiles.

Characteristics	Total (*n* = 1,168)	Quartiles of Lp(a)				*P* value
		Quartile 1 (*n* = 292)	Quartile 2 (*n* = 292)	Quartile 3 (*n* = 293)	Quartile 4 (*n* = 291)	
Age (years)	60.1 ± 11.1	58.4 ± 11.0	60.8 ± 11.1	61.3 ± 10.9	59.8 ± 11.1	0.006
Sex, *n* (%)						0.057
Male	960 (82.2)	242 (82.9)	247 (84.6)	247 (84.3)	224 (77.0)	
Female	208 (17.8)	50 (17.1)	45 (15.4)	46 (15.7)	67 (23.0)	
Vital signs						
Systolic blood pressure (mmHg)	121.7 ± 17.8	122.1 ± 17.5	124.2 ± 17.6	120.0 ± 18.1	120.5 ± 17.7	0.019
Diastolic blood pressure (mmHg)	73.0 ± 11.8	73.4 ± 11.0	74.0 ± 11.7	71.9 ± 12.7	72.6 ± 11.8	0.138
Heart rate (beats/min)	73.5 ± 10.5	73.5 ± 11.2	73.4 ± 9.9	73.6 ± 10.8	73.7 ± 10.3	0.99
Body mass index (kg/m^2^)	26.0 ± 10.7	26.0 ± 3.2	27.1 ± 20.6	25.5 ± 3.1	25.6 ± 3.5	0.24
NYHA class, *n* (%)						0.867
I	129 (11.0)	36 (12.3)	30 (10.3)	35 (11.9)	28 (9.6)	
II	628 (53.8)	152 (52.1)	154 (52.7)	158 (53.9)	164 (56.4)	
III	361 (30.9)	94 (32.2)	96 (32.9)	88 (30.0)	83 (28.5)	
IV	50 (4.3)	10 (3.4)	12 (4.1)	12 (4.1)	16 (5.5)	
Comorbidities, *n* (%)						
Atrial fibrillation	43 (3.7)	7 (2.4)	7 (2.4)	18 (6.1)	11 (3.8)	0.052
Hypertension	675 (57.8)	166 (56.8)	169 (57.9)	172 (58.7)	168 (57.7)	0.976
Diabetes	491 (42.0)	126 (43.2)	120 (41.1)	126 (43.0)	119 (40.9)	0.914
Hypercholesterolemia	877 (75.1)	216 (74.0)	215 (73.6)	213 (72.7)	233 (80.1)	0.152
History, *n* (%)						
Prior stroke	115 (9.8)	15 (5.1)	33 (11.3)	37 (12.6)	30 (10.3)	0.014
Prior MI	268 (22.9)	72 (24.7)	69 (23.6)	61 (20.8)	66 (22.7)	0.724
Prior PCI	111 (9.5)	30 (10.3)	26 (8.9)	31 (10.6)	24 (8.2)	0.74
Laboratory parameters						
White blood cell (109 /L)	8.0 ± 2.6	7.9 ± 2.7	8.0 ± 2.5	8.2 ± 3.0	7.8 ± 2.1	0.413
Red blood cell (109 /L)	4.5 ± 0.6	4.5 ± 0.6	4.5 ± 0.6	4.5 ± 0.6	4.5 ± 0.6	0.582
Hemoglobin (g/L)	139.1 ± 17.5	140.9 ± 18.0	139.1 ± 17.7	139.2 ± 16.7	137.1 ± 17.3	0.082
Platelet (109 /L)	225.6 ± 65.9	222.7 ± 64.9	219.1 ± 63.8	227.6 ± 66.3	233.1 ± 68.0	0.058
FBG (mmol/L)	7.4 ± 2.9	7.5 ± 2.9	7.3 ± 3.1	7.2 ± 2.8	7.4 ± 2.9	0.597
HbA1c (%)	6.9 ± 1.4	6.9 ± 1.4	6.8 ± 1.3	6.8 ± 1.5	6.9 ± 1.4	0.951
Creatinine (μmol/L)	76.9 [66.8, 88.3]	75.1 [66.1, 87.6]	78.1 [67.5, 87.6]	76.5 [67.4, 91.2]	77.9 [65.6, 89.0]	0.587
Blood nitrogen urea (mmol/L)	6.5 ± 2.9	6.2 ± 2.2	6.7 ± 3.3	6.7 ± 3.0	6.6 ± 3.0	0.121
eGFR (ml/min/1.73 m^2^)	88.2 ± 21.3	90.8 ± 20.0	87.6 ± 21.8	86.7 ± 21.9	87.7 ± 21.3	0.113
TG (mmol/L)	1.7 ± 1.0	1.8 ± 1.3	1.7 ± 1.1	1.6 ± 0.8	1.6 ± 0.8	0.006
TC (mmol/L)	4.0 ± 1.1	3.9 ± 1.0	4.0 ± 1.0	4.1 ± 1.1	4.2 ± 1.2	0.001
LDL-C (mmol/L)	2.4 ± 0.9	2.3 ± 0.9	2.4 ± 0.8	2.5 ± 1.0	2.6 ± 0.9	<0.001
HDL-C (mmol/L)	1.0 ± 0.2	1.0 ± 0.3	1.0 ± 0.2	1.0 ± 0.2	1.0 ± 0.2	0.645
Sodium (mmol/L)	138.8 ± 3.1	139.1 ± 3.0	138.7 ± 3.2	139.0 ± 3.1	138.7 ± 2.9	0.306
Potassium (mmol/L)	4.2 ± 0.5	4.1 ± 0.4	4.2 ± 0.5	4.2 ± 0.5	4.2 ± 0.5	0.279
BNP (pg/ml)	342.0 [153.0, 484.3]	301.0 [111.8, 460.3]	343.0 [175.3, 488.0]	358.0 [165.0, 507.0]	339.0 [166.5, 495.0]	0.043
hs-CRP(mg/L)	2.7 [1.0, 8.1]	2.5 [0.8, 6.1]	2.7 [1.0, 8.5]	3.1 [1.1, 9.5]	2.7 [0.9, 9.9]	0.048
Echocardiography						
Left atrial diameter (millimeter)	39.2 ± 5.0	39.0 ± 4.6	39.5 ± 4.9	39.0 ± 5.4	39.2 ± 5.2	0.583
LVDs (millimeter)	54.7 ± 6.9	54.6 ± 6.2	54.8 ± 6.8	54.7 ± 7.2	54.9 ± 7.3	0.935
LVDd (millimeter)	41.1 ± 7.8	41.0 ± 7.4	41.1 ± 7.6	41.0 ± 8.2	41.4 ± 8.0	0.911
LVEF (%)	40.8 ± 6.2	41.0 ± 5.9	40.8 ± 6.2	40.6 ± 6.8	40.9 ± 5.9	0.931
Medication use, *n* (%)						
Aspirin	1,162 (99.5)	292 (100.0)	290 (99.3)	292 (99.7)	288 (99.0)	0.338
Clopidogrel	932 (79.8)	241 (82.5)	219 (75.0)	236 (80.5)	236 (81.1)	0.115
Ticagrelor	235 (20.1)	51 (17.5)	73 (25.0)	56 (19.1)	55 (18.9)	0.109
Statins	1,161 (99.4)	290 (99.3)	290 (99.3)	291 (99.3)	290 (99.7)	0.935
Ezetimibe	301 (25.8)	74 (25.3)	72 (24.7)	73 (24.9)	82 (28.2)	0.75
CCB	149 (12.8)	33 (11.3)	38 (13.0)	36 (12.3)	42 (14.4)	0.714
Beta-blockers	705 (60.4)	179 (61.3)	181 (62.0)	168 (57.3)	177 (60.8)	0.665
ACEI	100 (8.6)	20 (6.8)	29 (9.9)	24 (8.2)	27 (9.3)	0.565
ARB	130 (11.1)	32 (11.0)	34 (11.6)	26 (8.9)	38 (13.1)	0.442
Diuretics	804 (68.8)	186 (63.7)	208 (71.2)	199 (67.9)	211 (72.5)	0.097
Angiographic data						
LM disease, *n* (%)	214 (18.3)	60 (20.5)	48 (16.4)	53 (18.1)	53 (18.2)	0.643
Three vessel disease, *n* (%)	669 (57.3)	159 (54.5)	171 (58.6)	177 (60.4)	162 (55.7)	0.452
Chronic total occlusion, *n* (%)	318 (27.2)	78 (26.7)	81 (27.7)	80 (27.3)	79 (27.1)	0.994
Diffuse lesion, *n* (%)	226 (19.3)	58 (19.9)	55 (18.8)	61 (20.8)	52 (17.9)	0.822
In-stent restenosis, *n* (%)	46 (3.9)	18 (6.2)	9 (3.1)	12 (4.1)	7 (2.4)	0.102
SYNTAX score	21.9 ± 7.8	22.2 ± 7.4	21.8 ± 8.0	21.8 ± 7.6	22.0 ± 8.1	0.911
Procedural results						
Target vessel territory, *n* (%)						
LM	201 (17.2)	58 (19.9)	45 (15.4)	48 (16.4)	50 (17.2)	0.523
LAD	895 (76.6)	223 (76.4)	216 (74.0)	225 (76.8)	231 (79.4)	0.494
LCX	759 (65.0)	182 (62.3)	189 (64.7)	197 (67.2)	191 (65.6)	0.655
RCA	805 (68.9)	198 (67.8)	207 (70.9)	205 (70.0)	195 (67.0)	0.719
Complete revascularization, *n* (%)	736 (63.0)	186 (63.7)	173 (59.2)	187 (63.8)	190 (65.3)	0.463
Number of stents	3.4 ± 1.5	3.3 ± 1.5	3.3 ± 1.4	3.4 ± 1.5	3.4 ± 1.5	0.312

Continuous variables were presented as mean ± SD or median (IQR). Categorical variables were presented as number (percentage). *P* values were calculated using analysis of variance, Kruskal–Wallis test or Chi-square test to compare differences in variables between different Lp(a) quartiles. NYHA, New York Heart Association; MI, myocardial infarction; PCI, percutaneous coronary intervention; FBG, fasting blood glucose; eGFR, estimated glomerular filtration rate; TC, total cholesterol; LDL-C, low-density lipoprotein cholesterol; HDL-C, high-density lipoprotein cholesterol; HbA1c, glycosylated hemoglobin A1c; BNP, B-natriuretic peptide; hs-CRP, high sensitivity C-reactive protein; LVDs, left ventricular end-systolic diameter; LVDd, left ventricular end-diastolic diameter; LVEF, left ventricular injection fraction; CCB, calcium channel blocker; ACEI, angiotensin-converting enzyme inhibitor; ARB, angiotensin receptor blocker; LM, left main artery; LAD, left anterior descending artery; LCX, left circumflex artery; RCA, right coronary artery; SYNTAX, synergy between PCI with taxus and cardiac surgery.

### 3.2 Incidence rates of adverse cardiovascular outcomes across Lp(a) quartiles

Table 2 illustrates that MACE incidence rose significantly across Lp(a) quartiles (*P* < 0.001), at 22.9%, 29.8%, 38.9%, and 46.4% for Quartiles 1 through 4, respectively. This trend was mainly driven by a higher incidence of all-cause mortality in higher Lp(a) quartiles (*P* < 0.001), which ranged from 11.6% in Quartile 1%–24.1% in Quartile 4. The rate of any revascularization also rose significantly with increasing Lp(a) levels (*P* = 0.021), from 8.9% in the lowest quartile to 17.2% in the highest. In contrast, no significant differences were found in non-fatal MI incidence across the Lp(a) quartiles (*P* = 0.202), despite a minor increase noted in the upper quartiles.

**Table 2:** Outcomes of patients stratified by Lp(a) quartiles.

Outcomes	Total (*n* = 1,168)	Quartiles of Lp(a)				*P* value
		Quartile 1 (*n* = 292)	Quartile 2 (*n* = 292)	Quartile 3 (*n* = 293)	Quartile 4 (*n* = 291)	
MACE, *n* (%)	403 (34.5)	67 (22.9)	87 (29.8)	114 (38.9)	135 (46.4)	<0.001
All-cause mortality	199 (17.0)	34 (11.6)	41 (14.0)	54 (18.4)	70 (24.1)	<0.001
Non-fatal MI	46 (3.9)	7 (2.4)	9 (3.1)	15 (5.1)	15 (5.2)	0.202
Any revascularization	158 (13.5)	26 (8.9)	37 (12.7)	45 (15.4)	50 (17.2)	0.021

Categorical variables were presented as number (percentage). *P* values were calculated using Chi-square test to compare differences in outcomes between different Lp(a) quartiles.

### 3.3 Cox regression analysis of Lp(a) levels and adverse cardiovascular outcomes

The Cox regression analysis (Table 3) revealed that elevated lipoprotein(a) concentrations were significantly associated with a higher risk of major adverse cardiovascular events in Model I (highest vs. lowest quartile: HR, 95% CI: 2.35, 1.75–3.15; P for trend <0.001). Model II, which adjusted for age and sex, showed that the highest quartile of lipoprotein(a) concentrations had a greater risk of major adverse cardiovascular events compared to the lowest quartile (HR, 95% CI: 2.33, 1.73–3.12, *P* < 0.001, P for trend <0.001). Model III, which included additional covariates, demonstrated that the group with the highest lipoprotein(a) concentrations remained independently associated with a higher risk of major adverse cardiovascular events (HR, 95% CI: 2.28, 1.69–3.07, *P* < 0.001, P for trend <0.001). Including lipoprotein(a) as a continuous variable in the models revealed that a 1-standard deviation increase in lipoprotein(a) concentrations was associated with a significantly elevated risk of major adverse cardiovascular events, with increments of 27%, 27%, and 26% in Models 1, 2, and 3, respectively. Moreover, Model 3 showed that lipoprotein(a) concentrations were positively associated with the risk of all-cause mortality (highest vs. lowest quartile: HR, 95% CI: 2.33, 1.54–3.54, *P* < 0.001, P for trend = 0.01) and any revascularization (highest vs. lowest quartile: HR, 95% CI: 2.18, 1.35–3.53, *P* = 0.002, P for trend = 0.001). However, the association between lipoprotein(a) concentrations and non-fatal MI was no longer statistically significant after adjusting for multiple factors (highest vs. lowest quartile: HR, 95% CI: 2.37, 0.95–5.88, *P* = 0.064, P for trend = 0.027). All Cox proportional hazards regression models involved in the study met the proportional hazards assumption (all Schoenfeld Test *p*-values > 0.05) (Supplementary Material).

**Table 3:** The association between Lp(a) and MACE.

	Model I			Model II			Model III		
	HR (95% CIs)	*P*	P for trend	HR (95% CIs)	*P*	P for trend	HR (95% CIs)	*P*	P for trend
MACE			<0.001			<0.001			<0.001
Quartile 1	1.0 (Ref)			1.0 (Ref)			1.0 (Ref)		
Quartile 2	1.34 (0.98–1.85)	0.070		1.30 (0.94–1.78)	0.111		1.24 (0.90–1.71)	0.201	
Quartile 3	1.82 (1.35–2.46)	<0.001		1.75 (1.29–2.37)	<0.001		1.75 (1.29–2.37)	<0.001	
Quartile 4	2.35 (1.75–3.15)	<0.001		2.33 (1.73–3.12)	<0.001		2.28 (1.69–3.07)	<0.001	
^a^Continuous	1.27 (1.17–1.37)	<0.001		1.27 (1.18–1.38)	<0.001		1.26 (1.16–1.37)	<0.001	
All-cause mortality			<0.001			<0.001			0.010
Quartile 1	1.0 (Ref)			1.0 (Ref)			1.0 (Ref)		
Quartile 2	1.25 (0.79–1.97)	0.341		1.22 (0.78–1.77)	0.382		1.19 (0.75–1.88)	0.459	
Quartile 3	1.70 (1.11–2.61)	0.015		1.66 (1.08–2.56)	0.021		1.68 (1.09–2.59)	0.018	
Quartile 4	2.40 (1.59–3.62)	<0.001		2.37 (1.57–3.57)	<0.001		2.33 (1.54–3.54)	<0.001	
^a^Continuous	1.30 (1.17–1.44)	<0.001		1.30 (1.17–1.45)	<0.001		1.29 (1.15–1.44)	<0.001	
Non-fatal MI			0.018			0.016			0.027
Quartile 1	1.0 (Ref)			1.0 (Ref)			1.0 (Ref)		
Quartile 2	1.33 (0.50–3.58)	0.568		1.22 (0.45–3.29)	0.691		1.16 (0.43–3.15)	0.772	
Quartile 3	2.31 (0.94–5.67)	0.067		2.11 (0.85–5.18)	0.106		2.02 (0.81–5.04)	0.132	
Quartile 4	2.53 (1.03–6.21)	0.043		2.55 (1.04–6.26)	0.041		2.37 (0.95–5.88)	0.064	
^a^Continuous	1.17 (0.91–1.51)	0.229		1.19 (0.92–1.54)	0.186		1.17 (0.89–1.52)	0.256	
Any revascularization			<0.001			0.001			0.001
Quartile 1	1.0 (Ref)			1.0 (Ref)			1.0 (Ref)		
Quartile 2	1.47 (0.89–2.43)	0.133		1.41 (0.85–2.33)	0.180		1.31 (0.79–2.19)	0.300	
Quartile 3	1.85 (1.14–3.00)	0.013		1.77 (1.09–2.87)	<0.001		1.75 (1.07–2.85)	0.025	
Quartile 4	2.24 (1.39–3.59)	0.001		2.21 (1.37–3.55)	0.001		2.18 (1.35–3.53)	0.002	
^a^Continuous	1.26 (1.11–1.42)	<0.001		1.27 (1.12–1.44)	<0.001		1.26 (1.11–1.44)	<0.001	

Models were derived from Cox proportional hazards regression analysis. Model I: unadjusted. Model II: adjusted for age, sex. Model III: adjusted for age, sex, heart rate, body mass index, NYHA class, prior PCI, TG, LDL-C, platelet, potassium, complete revascularization, diffuse lesion, chronic total occlusion, SYNTAX score, diffuse lesion, ARB, diuretics, statins.

^a^ HR per 1-SD increase in Lp(a).

### 3.4 Kaplan–Meier analysis of adverse cardiovascular outcomes across Lp(a) quartiles

**Figure 2:**
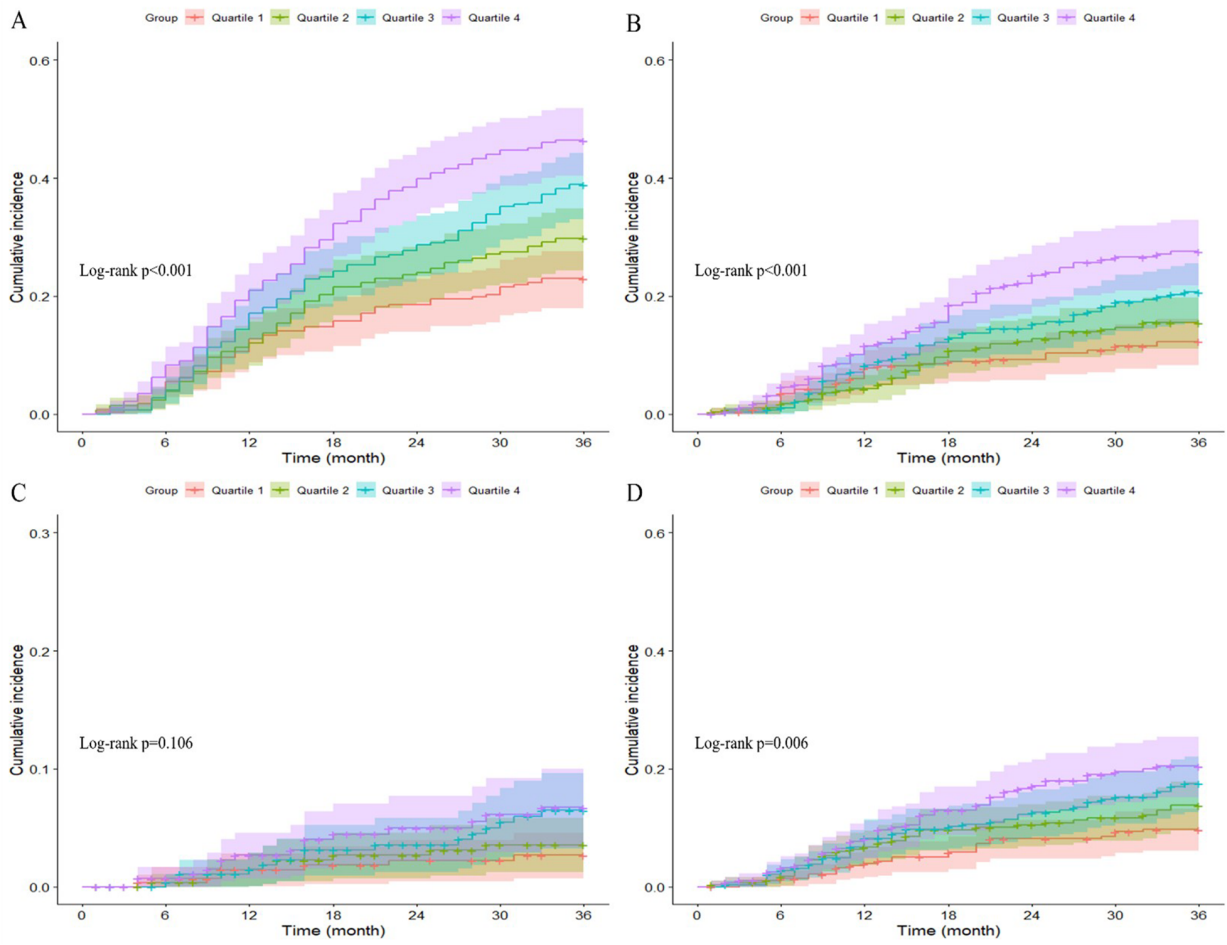
Kaplan–Meier curves showing the association between Lp(a) quartiles and MACE **(A)**, All-cause mortality **(B)**, non-fatal MI **(C)**, and any revascularization **(D)**.

The Kaplan-Meier curves in Figure 2 showed that patients in higher Lp(a) quartiles had a significantly higher cumulative incidence of major adverse cardiovascular events (log-rank test, *p* < 0.001), overall mortality (log-rank test, *p* < 0.001), and need for repeat revascularization (log-rank test, *p* = 0.006). Non-fatal MI rates trended higher with increasing Lp(a) quartiles, but this finding was not statistically significant (log-rank test, *p* = 0.106).

### 3.5 RCS analysis of nonlinear relationship between Lp(a) levels and MACE risk

**Figure 3:**
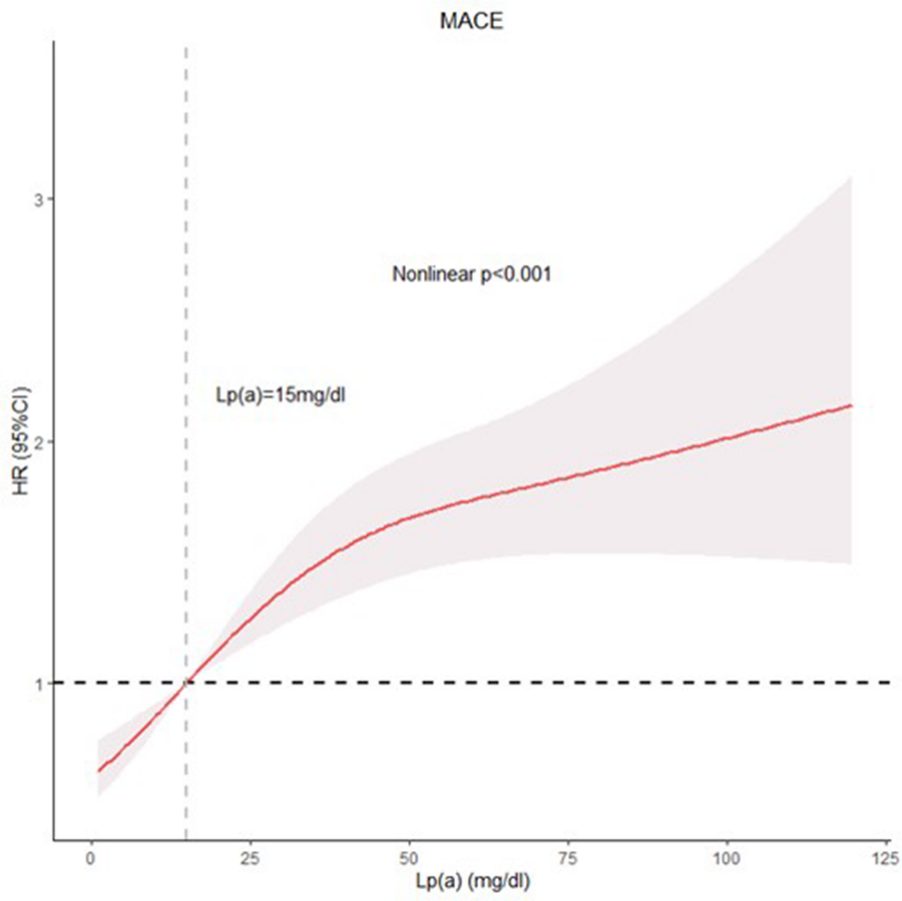
RCS model showing the associations of Lp(a) with MACE.

Figure 3 depicts the restricted cubic spline (RCS) model, which demonstrates a nonlinear association between Lp(a) concentrations and major adverse cardiovascular event risk (Nonlinear *p* < 0.001). The results, after accounting for potential confounding factors, showed that increasing Lp(a) levels were associated with a significant and gradual increase in the risk of major adverse cardiovascular events. This positive association suggested that elevated Lp(a) concentrations contribute to a higher likelihood of experiencing adverse outcomes.

### 3.6 Subgroup analysis

**Figure 4:**
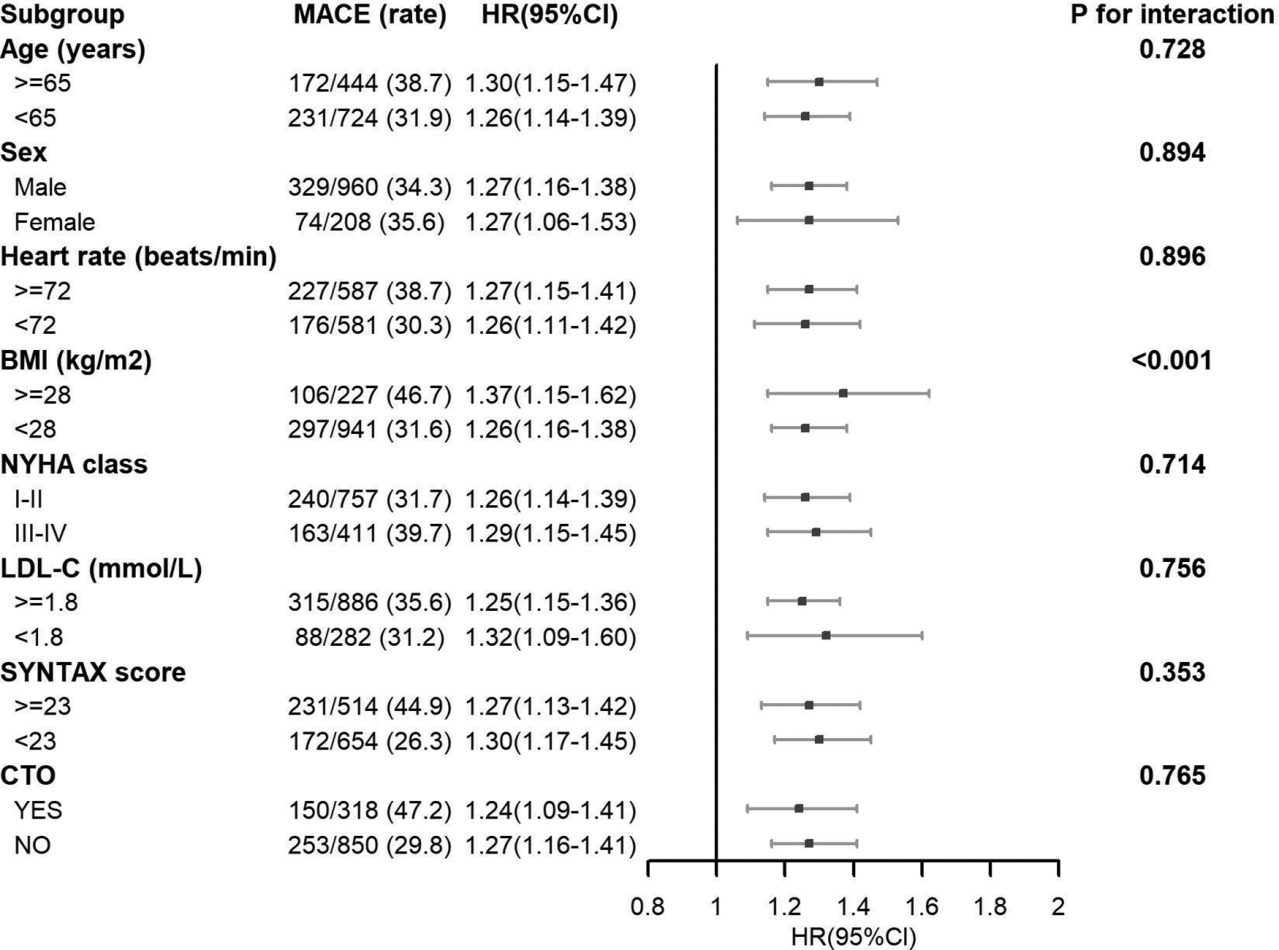
Subgroup analysis of associations between MACE and Lp(a).

For the majority of subgroups, such as sex, heart rate, NYHA class, LDL-C, SYNTAX score, and chronic total occlusion (CTO) of the coronary artery, no substantial interactions were noted. This suggested that the relationship between the study exposure and MACE risk was consistent across these subgroups (Figure 4). However, a significant interaction was found for BMI (P for interaction <0.001). Individuals having a BMI ≥ 28 kg/m^2^ showed a higher likelihood of experiencing MACE in contrast to individuals having a BMI < 28 kg/m^2^, as demonstrated by HR of 1.37 [1.15–1.41] and 1.26 [1.16–1.38], respectively. The association was more prominent in those with elevated BMI (According to the Chinese Expert Consensus on Medical Nutrition Therapy for Overweight/Obesity (2016 Edition), the cut-off value for overweight in China is set at a BMI of 28 kg/m^2^. This standard has been established based on the characteristics of the Chinese population and differs from internationally used criteria. Utilizing this localized cut-off point allows for a more accurate assessment of overweight and obesity status among the Chinese population, thereby providing a more appropriate reference standard for related health interventions and research).

## 4 Discussion

This study explored the relationship between Lp(a) concentrations and negative outcomes among individuals diagnosed with IHF. The findings demonstrated that elevated Lp(a) concentrations were independently linked to a higher likelihood of MACE, death from any cause, and the need for revascularization procedures. Moreover, the study identified a non-linear association between Lp(a) concentrations and the probability of experiencing MACE. Subgroup analyses showed a uniform relationship between the exposure variable and MACE probability across nearly all patient subgroups, with the exception of BMI, for which a notable interaction effect was detected. These findings provide insights into potential effect modifiers and can help guide future research and clinical decision-making.

Lp(a) is a distinct lipoprotein particle resulting from the covalent linkage of apoB-100 and apo(a) through a disulfide bridge (7). The uniqueness of Lp(a) lies in its apo(a) portion. The apo(a) component, encoded by the LPA gene on chromosome 6, exhibits substantial polymorphism, with numerous alleles contributing to considerable between-person differences in Lp(a) concentrations (7, 8). Extensive evidence from observational and genetic studies has consistently demonstrated that high Lp(a) concentrations are a standalone risk factor for ASCVD, encompassing CHD, ischemic cerebrovascular events, peripheral vascular disease, and valvular heart disease (9–11). A meta-analysis revealed that for every 50 mg/dl increment in Lp(a), the risk of CHD increases by approximately 30% (9). Furthermore, genetic studies have found that variations in the LPA gene locus are associated with CHD risk, further confirming the causal relationship between the two (12). Currently, Lp(a) has been recommended by the European Atherosclerosis Society guidelines as an emerging risk factor for assessing ASCVD risk (13).

Moreover, numerous studies have investigated the influence of Lp(a) on the extended prognosis of individuals with coronary heart disease following PCI. Research involving 3,313 individuals who underwent PCI discovered that those with elevated Lp(a) concentrations faced a substantially greater likelihood of MACE and cardiac mortality in comparison to those with reduced Lp(a) concentrations following a three-year follow-up period (14). Zhu et al. (15) additionally discovered that PCI individuals with elevated Lp(a) concentrations experienced increased rates of platelet aggregation and ischemic incidents during the post-procedural monitoring period. Furthermore, research on individuals with AMI implied that elevated Lp(a) concentrations could be a risk factor for MACE during hospitalization (16). A 36-month study that monitored 506 individuals with coronary heart disease and left ventricular systolic dysfunction demonstrated that the likelihood of all-cause mortality was 2.98 times greater in those with elevated Lp(a) concentrations compared to those with reduced Lp(a) concentrations (17). These studies highlight the important role of Lp(a) in the secondary prevention of ASCVD (18, 19).

However, investigations into the significance of lipoprotein(a) in individuals with ischemic heart failure (IHF) are still scarce. Considering the pivotal function of lipoprotein(a) in atherosclerotic cardiovascular disease (ASCVD), it is postulated that this lipoprotein might impact the prognosis of individuals with IHF by influencing the advancement of the condition. Contemporary research has investigated the association of diverse biological markers with unfavorable results in individuals diagnosed with IHF. Indicators of inflammation, including red cell distribution width (RDW) (22) and systemic inflammation response index (SIRI) (23), along with nutritional status parameters like geriatric nutritional risk index (GNRI) (24), have shown substantial correlations with the likelihood of major adverse cardiovascular events (MACE) in this cohort. This research concentrates on lipoprotein(a), a biological marker that has recently garnered significant interest, to examine its relationship with prognosis in individuals diagnosed with IHF. To minimize the impact of possible confounding variables, the researchers utilized Cox multivariate regression analysis. This methodology allowed the identification of lipoprotein(a) as a standalone risk factor for MACE in individuals with IHF, while simultaneously unveiling a non-linear direct relationship between lipoprotein(a) concentrations and the likelihood of MACE incidents. The results implied that lipoprotein(a) might function as a predictive biological marker for risk categorization in individuals diagnosed with IHF, and that decreasing lipoprotein(a) concentrations could potentially provide advantages to these individuals. This observation underscored the importance of monitoring Lp(a) levels in addition to focusing on LDL, TC, and other serum lipid parameters in clinical practice (25, 26). This investigation offered possible evidence-based backing for the management of lipoprotein(a) in this particular patient group and emphasized the necessity for healthcare professionals to regard lipoprotein(a) as a significant indicator in the risk evaluation and management of individuals with IHF.

Obesity is widely regarded as a significant contributor to heart-related ailments. However, in certain chronic diseases, the “obesity paradox” has been observed, wherein overweight and obese patients experience improved cardiovascular outcomes compared to their leaner counterparts (27–30). Based on this concept, our study conducted a subgroup analysis of BMI and found a significant interaction between BMI and the association of the study exposure with MACE risk in IHF patients, with a stronger association observed in patients with higher BMI (≥28 kg/m^2^). The fundamental reason might be linked to the inflammation-promoting condition connected with obesity, which plays a role in the onset and advancement of plaque buildup in arteries (31, 32). Moreover, obesity is often accompanied by other metabolic disorders, such as insulin resistance and dyslipidemia (33, 34). These elements may interplay with heightened Lp(a) concentrations to additionally elevate the likelihood of adverse cardiovascular events in IHF patients with increased BMI. This intriguing discovery implied that in healthcare settings, particular focus should be given to individuals with obesity, including regular surveillance of Lp(a) concentrations and active intervention to diminish the likelihood of unfavorable outcomes in this patient population. Clinicians should have been aware of the potential interaction between obesity and elevated Lp(a) levels, which might have synergistically contributed to increased cardiovascular risk in IHF patients. Regular screening of Lp(a) levels in obese IHF patients, along with comprehensive management of obesity and associated metabolic disorders, might have helped improve patient outcomes. Adopting healthy behavior adjustments, including shedding excess pounds, altering eating habits, and engaging in more exercise, alongside medical therapies aimed at Lp(a) and additional risk elements, might have proven to be a potent approach to lessen the elevated risk noted in this subset of individuals.

### 4.1 Strengths

First, it was one of the few studies to explore the association between Lp(a) levels and adverse outcomes specifically in patients with IHF, providing novel insights into the prognostic significance of Lp(a) in this population. Second, the study utilized a relatively large cohort of 1,168 IHF patients, specifically from the Chinese population, enhancing the statistical power, generalizability, and cultural relevance of the findings. Third, the use of Cox proportional hazards models and RCS analysis allowed for a detailed examination of both linear and nonlinear relationships between Lp(a) levels and MACE, offering a more comprehensive understanding of the risk factors. Lastly, the inclusion of subgroup analyses, particularly focusing on BMI, highlighted important interactions and potential effect modifiers, contributing to more personalized risk assessments and management strategies in clinical practice.

### 4.2 Limitation

(1) As an observational study, the results may be affected by potential confounding factors and selection bias. Although we adjusted for multiple variables, residual confounding cannot be excluded. (2) Due to the retrospective nature of our study, information on alcohol consumption, smoking, physical exercise, and diet was not available. (3) As the research was performed at one institution, the external validity of the results may be limited. To corroborate the findings, investigations involving multiple sites and a broader patient cohort are necessary. (4) Lp(a) levels were measured only once at baseline. Given the intra-individual variability of Lp(a), repeated measurements over time would provide a more accurate assessment of Lp(a) exposure. (5) The study did not explore the potential mechanisms underlying the association between Lp(a) and adverse outcomes in ischemic heart failure. Additional studies are required to clarify the underlying biological mechanisms. (6) The impact of Lp(a) lowering therapies on the prognosis of IHF patients remains unclear. To assess the therapeutic impact of Lp(a) reduction in these patients, randomized controlled trials are essential.

## 5 Conclusion

In individuals with IHF, Lp(a) independently predicted MACE, all-cause mortality, and any revascularization. Furthermore, a curvilinear association was noted between Lp(a) concentrations and MACE occurrence. The analysis additionally identified a notable interplay with BMI, indicating that the link between higher Lp(a) and greater MACE risk was stronger in those with obesity. These results could offer new perspectives to improve risk assessment and individualized management approaches for people with IHF.

## Data Availability

The raw data supporting the conclusions of this article will be made available by the authors, without undue reservation.
