# Supplementary material for: Lipoprotein(a) as a novel biomarker for predicting adverse outcomes in ischemic heart failure
Source: Front Cardiovasc Med. 2024 Sep 5;11:1466146. doi: 10.3389/fcvm.2024.1466146 (PMC11410592; doi:10.3389/fcvm.2024.1466146)
Supplement: Supplementary file 1 [file Datasheet1.pdf]

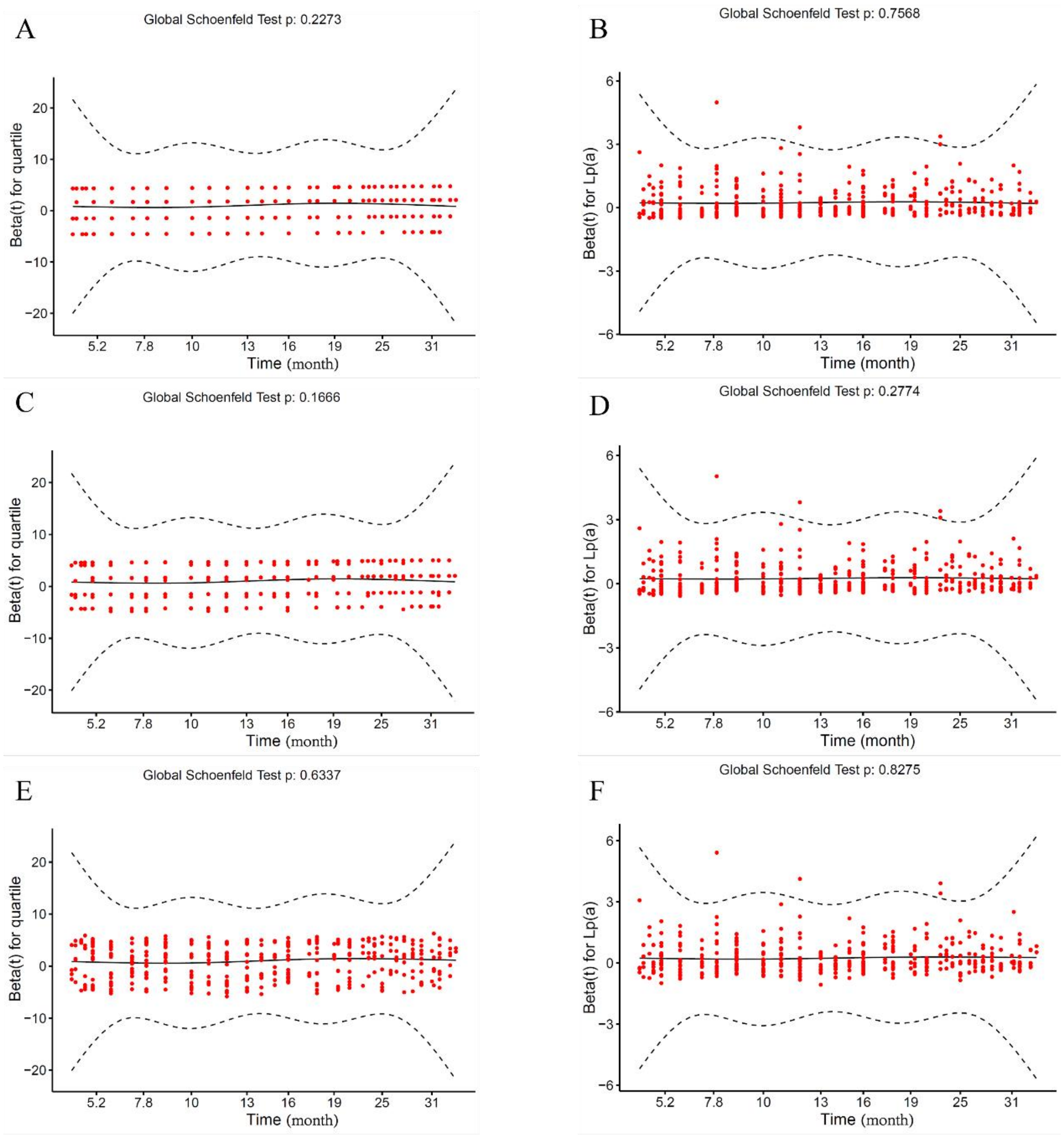

Figure S1. Standardized Schoenfeld Residual Plots for Testing the Proportional Hazards Assumption.

(A) Model I with Lp(a) as a grouped variable.

(B) Model I with Lp(a) as a continuous variable.

(C) Model II with Lp(a) as a grouped variable.

(D) Model II with Lp(a) as a continuous variable.

(E) Model III with Lp(a) as a grouped variable.

(F) Model III with Lp(a) as a continuous variable.
